# Comment on: The m6A Reader IGF2BP2 Regulates Macrophage Phenotypic Activation and Inflammatory Diseases by Stabilizing TSC1 and PPAR*γ*

**DOI:** 10.1002/advs.202104372

**Published:** 2022-01-17

**Authors:** Hanna S. Schymik, Charlotte Dahlem, Ahmad Barghash, Alexandra K. Kiemer

**Affiliations:** ^1^ Department of Pharmacy Pharmaceutical Biology Saarland University Saarbruecken 66123 Germany; ^2^ School of Electrical Engineering and Information Technology German Jordanian University Amman 11180 Jordan

**Keywords:** human macrophages, IFN*γ* Ι IGF2BP2, LPS, macrophage polarization, mouse macrophages, species difference

## Abstract

Recently, first insights into the regulation and the role of the RNA‐binding protein IMP2 in macrophage activation have been published by Wang et al. This study addresses differences in the regulation of IMP2 between the human and murine system. While the expression of IMP2 in anti‐inflammatory macrophages is synchronous in mice and men, IMP2 expression is regulated differently in inflammatory macrophages.

## Graphical abstract

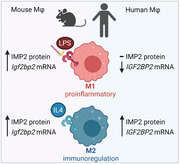

With recently published data in *Advanced Science*, Wang et al. for the first time report a role of the insulin‐like growth factor 2 (IGF2) mRNA‐binding protein 2 (IGF2BP2/IMP2/VICKZ2) in macrophage activation.^[^ ^1^ ^]^

As a highly conserved RNA binding protein, IGF2BP2 plays an essential role in the translation, stabilization, localization, modification, and processing of various mRNA targets and, accordingly, influences physiological and pathophysiological processes in the context of metabolism and malignancy.^[^ ^2^, ^3^, ^4^ ^]^ However, beyond the knowledge on its promotion of inflammatory processes in fatty liver disease and autoantibody‐induced glomerulonephritis, little is known about the role of IGF2BP2 in inflammatory conditions.^[^ ^5^, ^6^ ^]^

Wang et al. suggest that a loss of *Igf2bp2* leads to an enhanced inflammatory M1 phenotype of murine macrophages by stabilizing *Tsc1* and *Pparg*.^[^ ^1^ ^]^

Here, we would like to report our data on the regulation of *IGF2BP2* in polarized human monocyte‐derived macrophages (HMDMs) and our analysis of *IGF2BP2* expression levels in various inflammatory conditions in the human system. These data suggest a more complex view of IGF2BP2 in inflammation and, to some extent, contradict the conclusions of Wang et al.

In HMDMs treated with bacterial lipopolysaccharide (LPS) for up to 24 h, we found a significant reduction in *IGF2BP2* mRNA expression (**Figure** **1**A), while protein levels were not changed (Figure 1B). Similarly, analysis of a proteomics dataset from the human macrophage‐like THP‐1 cell line polarized toward M1 by LPS and IFN*γ* for either 6 h or 48 h revealed no difference in IGF2BP2 protein levels (Figure S1, Supporting Information).

**Figure 1:**
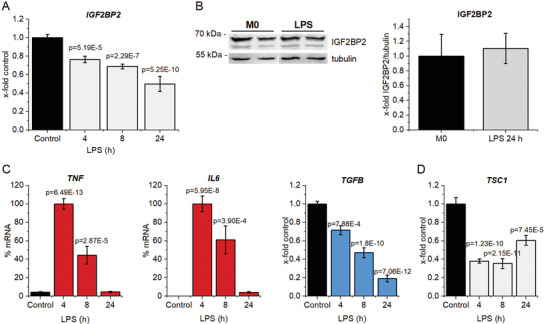
*IGF2BP2 in activated human macrophages*. Human monocyte‐derived macrophages (HMDMs) were treated with 100 ng mL^−1^ LPS for the indicated time. IGF2BP2 expression was determined on A) gene and B) protein level (24 h). Expression data were normalized to *ACTB* or tubulin, respectively, and are represented as means ± SEM (x‐fold control). C) *TNF*, *IL6*, *TGFB, and* D) *TSC1* were quantified upon LPS treatment. Data are represented as means ± SEM (x‐fold 4 h LPS for *TNF* and *IL6*; x‐fold control for *TGFB* and *TSC1*). Statistical analysis was performed by ANOVA analysis followed by Bonferroni's post hoc test; *n* = 4, triplicates (4 h; 8 h LPS); *n* = 2, triplicates (24 h).

At early activation time points, *IGF2BP2* reduction in HMDMs was accompanied by the induction of the inflammatory cytokines TNF and IL6 and a reduction of the anti‐inflammatory cytokine TGFB (Figure 1C). Importantly, prolonged LPS stimulation for 24 h represents a rather desensitized macrophage activation state, as confirmed by the absence of *TNF* and *IL6* expression (Figure 1C).^[^ ^7^ ^]^ These findings are in contrast to the IGF2BP2 mRNA and protein induction upon LPS treatment of murine bone marrow‐derived macrophages (BMDMs), as demonstrated by Wang et al.^[^ ^1^ ^]^

The expression of *TSC1*, which was suggested by Wang et al. to facilitate IGF2BP2 action, correlated with *IGF2BP2* and was distinctly reduced upon LPS treatment (Figure 1D).

To exploit whether this decline of *IGF2BP2* expression during inflammation is specific for LPS‐facilitated inflammatory cell activation, we analyzed *IGF2BP2* expression in several publicly available data sets, in which human blood, monocytes, or macrophages were infected with either Gram‐positive or Gram‐negative bacteria or viruses, causing various types of Toll‐like receptor (TLR) activation.^[^ ^8^, ^9^ ^]^ The presence of acute inflammation was confirmed by an elevated *TNF* expression of the specific samples. Our analyses demonstrate a reduced *IGF2BP2* levels in all models of acute inflammation, although statistical significance was not reached in all of them (**Figure** **2**). Taken together, in contrast to the murine data from Wang et al., our analyses of human samples suggest a downregulation of *IGF2BP2* during acute inflammation.

**Figure 2:**
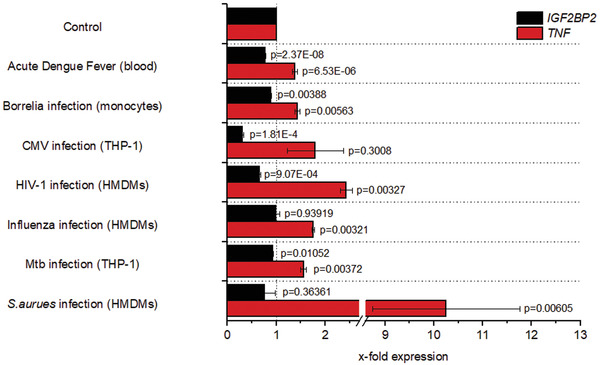
*Expression levels of IGF2BP2 after infection. IGF2BP2* and *TNF* expression levels were compared with the respective healthy non‐infected controls, which were set to 1. Expression analysis of *IGF2BP2* and *TNF* in whole blood samples of patients with acute dengue fever (GSE51808, *n* = 9 (healthy), 18 (infected)); of human isolated monocytes infected with *Borrelia burgdorferi* (20 h) (GSE103483, *n* = 3 (control, infected)); of the macrophage‐like THP‐1 cell line infected with *Cytomegalovirus* (24 h) (GSE141236, *n* = 3 (control, infected)); of HMDMs infected with *HIV‐1*‐BaL‐HAS (18 h) (GSE158434, *n* = 3 (control, infected)); of HMDMs infected with Influenza H5N1 (6 h) (GDS3595, *n* = 3 (control, infected)); of the THP‐1 cell line infected with *Mycobacterium tuberculosis* (Mtb) H37Rv (GDS4781, *n* = 3 (control, infected)); of HMDMs infected with *Staphylococcus aureus* (*S. aureus*) (8 h) (GDS4931 *n* = 5 (control, infected)). Data are represented as means ± SEM. *P*‐values were calculated by Student's t‐test for each dataset.

In addition to their role in acute inflammation, macrophages can exhibit a broad polarization spectrum, with the M1 and M2 phenotypes representing two extreme ends. Classically activated (M1) macrophages act as effector cells in the Th1 response and are usually generated in vitro by the stimulation with LPS and IFN*γ*. Different M2 macrophage subsets can be generated using IL4 or IL10 for polarization.^[^ ^10^ ^]^

The analysis of *IGF2BP2* expression revealed that human M1 macrophages display no alterations in expression, while M2 polarized human macrophages show a significant induction on gene and protein level (**Figure** **3**A,B). These data on human M2 macrophages are consistent with the data of Wang et al. as well as the observation that treatment by IFN*γ* alone induces *IGF2BP2* in both human and murine macrophages (see Figure S2, Supporting Information). IFN*γ* and LPS typically synergize and result in a pro‐inflammatory macrophage “super‐activation”. IFN*γ* treatment by itself induces different signaling than LPS: It activates the STAT1 transcription factor and induces the opening of chromatin. This so‐called priming not necessarily induces a transcriptional activity but rather “bookmarks” inflammatory genes for LPS‐induced gene transcription.^[^ ^11^ ^]^ The observation that IFN*γ* treatment by itself does in fact induce *IGF2BP2* warrants future investigations.

**Figure 3:**
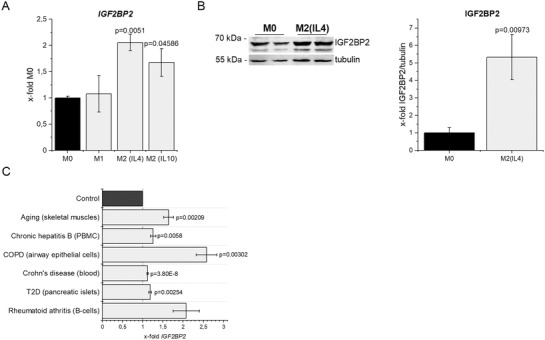
*IGF2BP2 expression in alternatively activated macrophages and various chronic inflammatory diseases*. HMDMs were polarized for 24 h and IGF2BP2 expression was determined on A) gene and B) protein level. Expression data were normalized to *18S* or tubulin, respectively, and are represented as means ± SEM (x‐fold M0). Statistical analysis was performed by ANOVA analysis followed by Bonferroni's post hoc test, *n* = 2, triplicates (M2 (IL‐4)), *n* = 4, duplicates (M2 (IL‐10)). C) *IGF2BP2* expression levels were compared with the respective healthy controls, which were set to 1. Expression analysis of *IGF2BP2* of old skeletal muscles (GDS5218, *n* = 7 (young) *n* = 6 (old) female); peripheral blood mononuclear cells (PBMCs) of patients with chronic hepatitis B (GSE114783, *n* = 10 (chronic hepatitis B), *n* = 3 (healthy)); airway epithelial cells of patients with COPD (GSE5058, *n* = 6 (COPD), *n* = 12 (healthy)); of whole blood samples of patients with Crohn's diseases (GSE126124: *n* = 40 (Crohn's diseases), *n* = 32 (healthy)); of pancreatic islets of patients with type 2 diabetes (T2D, GDS3882, *n* = 6 (Diabetes), *n* = 6 (healthy)); of B‐cells of patients with rheumatoid arthritis (GSE4588, *n* = 7 (arthritis), *n* = 9 (healthy)). Data are represented as means ± SEM. *P*‐values were calculated by Student's *t*‐test for each dataset.

The anti‐inflammatory M2 phenotype plays a role in chronic inflammation by supporting tissue remodeling and repair.^[^ ^12^ ^]^ In line with the findings by Wang et al., *IGF2BP2* is elevated in conditions of chronic inflammation, such as aging, chronic hepatitis B, obstructive pulmonary disease (COPD), Crohn's diseases, type 2 diabetes, and rheumatoid arthritis (Figure 3C).

In conclusion, our data suggest that *IGF2BP2* is differentially regulated in acute inflammation in humans versus mice. Mouse macrophages in general show a limited responsiveness toward LPS, which has been suggested to be due to a rapid induction of negative feedback regulators.^[^ ^13^ ^]^ Since *IGF2BP2* is consistently induced in both murine and human M2 macrophages, which are desensitized toward activation, the induction of IGF2BP2 in murine macrophages by LPS might contribute to the attenuated responsiveness of murine macrophages.

In contrast to LPS treatment, there is a high consistency of *IGF2BP2* expression in chronic inflammatory conditions between mouse and man. Future studies are required to gain insight into the role of IGF2BP2 at different stages of inflammation.

## Conflict of Interest

The authors declare no conflict of interest.

## Supporting information

Supporting Information

## Data Availability

Research data are not shared.
