# Supplementary material for: Comment on: The m6A Reader IGF2BP2 Regulates Macrophage Phenotypic Activation and Inflammatory Diseases by Stabilizing TSC1 and PPARγ
Source: Adv Sci (Weinh). 2022 Jan 17;9(8):2104372. doi: 10.1002/advs.202104372 (PMC8922093; doi:10.1002/advs.202104372)
Supplement: Supplementary file 1 — Supporting Information [file ADVS-9-2104372-s001.pdf]

ADVANCED SCIENCE
Open Access

Supporting Information

for *Adv. Sci.*, DOI 10.1002/advs.202104372

Comment on: The m6A Reader IGF2BP2 Regulates Macrophage Phenotypic Activation and Inflammatory Diseases by Stabilizing TSC1 and PPARγ

*Hanna S. Schymik, Charlotte Dahlem, Ahmad Barghash and Alexandra K. Kiemer**

ADVANCED SCIENCE
Open Access

Supporting Information

for *Adv. Sci.,* DOI: 10.1002/advs.202104372

Comment on: The m6A Reader IGF2BP2 Regulates Macrophage Phenotypic Activation and Inflammatory Diseases by Stabilizing TSC1 and PPARγ

*Hanna S. Schymik, Charlotte Dahlem, Ahmad Barghash, Alexandra K. Kiemer**

Comment on: The m6A Reader IGF2BP2 Regulates Macrophage Phenotypic Activation and Inflammatory Diseases by Stabilizing TSC1 and PPARγ

*Hanna S. Schymik, Charlotte Dahlem, Ahmad Barghash, Alexandra K. Kiemer**

H. S. Schymik, Dr. C. Dahlem, Prof. Dr. A. K. Kiemer
Department of Pharmacy
Pharmaceutical Biology
Saarland University
Saarbruecken, 66123, Germany
E-mail: pharm.bio.kiemer@mx.uni-saarland.de

Dr. A. Barghash
School of Electrical Engineering and Information Technology
German Jordanian University
Amman 11180, Jordan

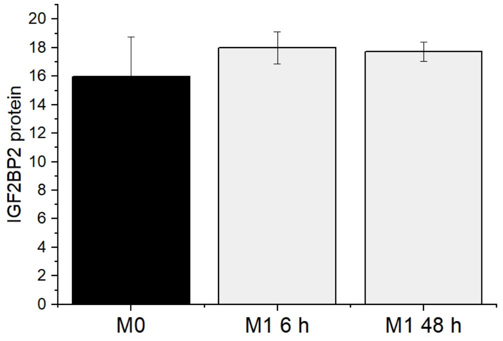

Supplementary Figure 1: *IGF2BP2 protein in M1-polarized THP-1.* Expression analysis of IGF2BP2 protein levels from the proteomics data set PXD017391 (ProteomeXchange) in differentiated THP-1 cells (n=3). For differentiation THP-1 were treated for 48 h with 100 ng/ml phorbol-12-myristate-13-acetate (PMA). THP-1 were polarized with 1 µg/ml LPS and 20 ng/ml IFNγ over 6 h and 48 h. Data are represented as means ± SEM.

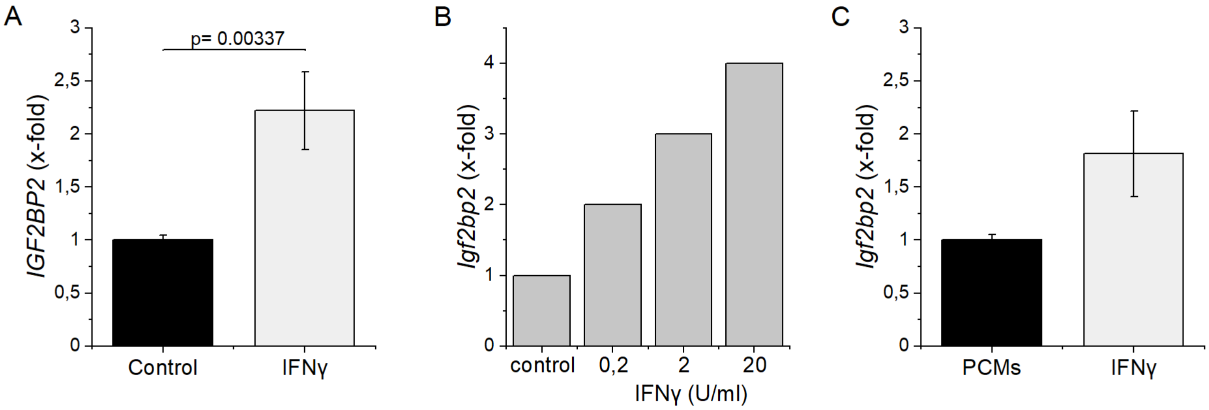

Supplementary Figure 2: *IGF2BP2 in IFNγ -treated macrophages.* (**A**) Human monocyte-derived macrophages (HMDMs) were treated with 20 ng/ml IFNγ for 24 h. *IGF2BP2* expression was normalized to *18S* and is demonstrated as means ± SEM (x-fold control). P-value was calculated by Student's t-test; n=3, triplicates. (**B**) *Igf2bp2* levels in murine bone marrow-derived macrophages treated with IFNγ at the indicated concentrations for eight hours (GSE116364). (**C**) *Igf2bp2* expression was determined in peritoneal cavity macrophages, from mice which had been injected with IFNγ (8 U) (mice were sacrificed two h after injection, GSE110549). Data are represented as means ± SEM (x-fold control). P-value was calculated by Student's t-test; n=3, triplicates. (**A, B, C**) Data were compared to untreated control cells, which were set to 1.

Methods

HMDM isolation and differentiation

Human monocyte-derived macrophages (HMDMs) were isolated and differentiated as described before and treated with 100 ng/ml LPS (Ultrapure LPS from *Escherichia coli* K12 #tlrl-peklps) for the indicated time.[14] For polarization HMDMs were treated with 20 ng/mL recombinant IFNγ (Miltenyi #130-096-484) and 100 ng/mL LPS (Ultrapure LPS from *Escherichia coli* K12 #tlrl-peklps) for M1 polarization; either 20 ng/mL IL4 (Miltenyi #130-093-921) or IL10 (Miltenyi #130-093-948) for M2 polarization; or left without further supplementation for M0 for 24 hours.

Untreated HMDMs served as controls. Permission to use human material for primary cell isolation was obtained from the local ethics committee. (State Medical Board of Registration, Saarland, Germany; permission no. 173/18).

qPCR

RNA was isolated using the High Pure RNA Isolation Kit (#11828665001; Roche) according to the manufacturer's instructions. Quantitative RT-PCR (qPCR) was performed as described before.[15] Gene expression was normalized to *ACTB* or *18S* respectively.

**Primer**	**Sequence**
hu_18S_fw	AGGTCTGTGATGCCCTTAGA
hu_18S_rev	GAATGGGGTTCAACGGGTTA
hu_ACTB_fw	TGCGTGACATTAAGGAGAAG
hu_ACTB_rev	GTCAGGCAGCTCGTAGCTCT
hu_CXCL8_fw	GAGAAGTTTTTGAAGAGGGCTGA
hu_CXCL8_rev	GCTTGAAGTTTCACTGGCATCT
hu_IGF2BP2_fw	GTTCCCGCATCATCACTCTTAT
hu_IGF2BP2_rev	GAATCTCGCCAGCTGTTTGA
hu_IL6_fw	ACATCCTCGACGGCATCTCA
hu_IL6_rev	TCACCAGGCAAGTCTCCTCATT
hu_TGFB_fw	GTGGACATCAACGGGTTCACT
hu_TGFB_rev	CGCACGCAGCAGTTCTTCTC
hu_TNF_fw	CTCCACCCATGTGCTCCTCA
hu_TNF_rev	CTCTGGCAGGGGCTCTTGAT
hu_TSC1_fw	AGAGCCACATGACAAGCACC
hu_TSC1_rev	GGATAAACGAGTGGCGGCTT

Proteomic analysis

The proteomic dataset PXD017391 was acquired from the PRIDE archive from the ProteomeXchange Consortium.[16-18] The proteomic analysis was performed with the MaxQuant quantitative proteomics software package in high performance workstation environment.[19]

Statistical Analysis

Data were normalized to the respective controls as indicated, followed by Grubb's test for outlier identification. The data are represented as means ± SEM (standard error of the 3mean) of at least 3 independent experiments performed in replicates, if not indicated otherwise. Statistical differences between two groups were calculated using a two-tailed Student's t-test; one-way Analysis of Variance (ANOVA) analysis followed by Bonferroni's post-hoc test was used for statistical comparison of more than two groups. All tests are two-sided and differences were considered statistically significant when p-values were less than 0.05. Calculations were performed using the OriginPro® 2020 software. Data analysis for proteomics was performed using R and plotted using Tableau. Fold-changes for protein abundance were calculated using the mean in each group.
